# Phosphorylation of GAPVD1 Is Regulated by the PER Complex and Linked to GAPVD1 Degradation

**DOI:** 10.3390/ijms22073787

**Published:** 2021-04-06

**Authors:** Hussam Ibrahim, Philipp Reus, Anna Katharina Mundorf, Anna-Lena Grothoff, Valerie Rudenko, Christina Buschhaus, Anja Stefanski, Niklas Berleth, Björn Stork, Kai Stühler, Faiza Kalfalah, Hans Reinke

**Affiliations:** 1 Institute of Clinical Chemistry and Laboratory Diagnostics, Medical Faculty, University of Düsseldorf, 40225 Düsseldorf, Germany; hussam.ibrahim@med.uni-duesseldorf.de (H.I.); Philipp.reus@ime.fraunhofer.de (P.R.); anmun102@hhu.de (A.K.M.); angro106@hhu.de (A.-L.G.); valerie.rudenko@hhu.de (V.R.); Christina.Buschhaus@med.uni-duesseldorf.de (C.B.); faiza.khalfalah@med.uni-duesseldorf.de (F.K.); 2 Biologisch-Medizinisches Forschungszentrum, Molecular Proteomics Laboratory, University of Düsseldorf, 40225 Düsseldorf, Germany; Anja.stefanski@uni-duesseldorf.de (A.S.); kai.stuehler@uni-duesseldorf.de (K.S.); 3 Institute of Molecular Medicine I, Medical Faculty, University of Düsseldorf, 40225 Düsseldorf, Germany; Niklas.Berleth@uni-duesseldorf.de (N.B.); bjoern.stork@uni-duesseldorf.de (B.S.)

**Keywords:** circadian clock, PER complex, GAPVD1, protein phosphorylation, protein degradation

## Abstract

Repressor protein period (PER) complexes play a central role in the molecular oscillator mechanism of the mammalian circadian clock. While the main role of nuclear PER complexes is transcriptional repression, much less is known about the functions of cytoplasmic PER complexes. We found with a biochemical screen for PER2-interacting proteins that the small GTPase regulator GTPase-activating protein and VPS9 domain-containing protein 1 (GAPVD1), which has been identified previously as a component of cytoplasmic PER complexes in mice, is also a bona fide component of human PER complexes. We show that in situ GAPVD1 is closely associated with casein kinase 1 delta (CSNK1D), a kinase that regulates PER2 levels through a phosphoswitch mechanism, and that CSNK1D regulates the phosphorylation of GAPVD1. Moreover, phosphorylation determines the kinetics of GAPVD1 degradation and is controlled by PER2 and a C-terminal autoinhibitory domain in CSNK1D, indicating that the regulation of GAPVD1 phosphorylation is a novel function of cytoplasmic PER complexes and might be part of the oscillator mechanism or an output function of the circadian clock.

## 1. Introduction

Circadian clocks are endogenous mechanisms that drive rhythmicity of physiology and behavior, enabling light-sensitive organisms to anticipate daily recurring changes in the course of the day–night cycle. The mammalian circadian system is synchronized to the environment by a light-responsive master clock in the hypothalamus that transmits timing information to peripheral oscillators via neuronal and endocrine pathways [1]. Rhythmicity of the master clock and peripheral clocks is generated by a molecular oscillator mechanism that exists in basically all cells of the body. The central working principle of the molecular oscillator is a negative feedback loop of the transcriptional repressor proteins period (PER) and cryptochrome (CRY). Their expression is driven by heterodimers of the transcriptional activator aryl hydrocarbon receptor nuclear translocator-like protein 1 (ARNTL/BMAL1) with circadian locomotor output cycles kaput (CLOCK) or its paralog neuronal PAS (Per-Arnt-Sim) domain protein 2 (NPAS2). When PER and CRY proteins reach a critical concentration, they repress transcription at their own gene loci so that a new cycle can begin [2].

PER and CRY proteins function as integral components of PER complexes, highly dynamic protein assemblies that work in the core feedback loop of the molecular clock. Nuclear and cytoplasmic PER complexes differ in their protein compositions, which reflect diverging functions in different cellular compartments. Cytoplasmic PER complexes exist in two variants; a smaller complex that contains the core components PER1/2, CRY1/2, and casein kinase 1 delta (CSNK1D), and a bigger complex that additionally contains PER3 and GTPase-activating protein and VPS9 domain-containing protein 1 (GAPVD1) [3]. Nuclear PER complexes are much larger and contain, in addition to the core components, a wide variety of proteins with mainly gene regulatory functions, such as RNA helicases, chromatin regulators, and transcriptional co-repressors [4,5,6]. While nuclear PER complexes are predominantly involved in the negative regulation of transcription, it is largely unknown if cytoplasmic PER complexes are essentially assembly intermediates towards a complex that is competent for nuclear entry or if cytoplasmic PER complexes contribute significantly to non-transcriptional functions of the molecular oscillator.

The circadian clock regulates a wide variety of output functions by controlling rate limiting steps of cellular pathways. For this purpose, clock proteins possess additional regulatory functions outside the oscillator mechanism that establish molecular links to cellular physiology, in general, via the transcriptional regulation of individual sets of target genes [7]. However, accumulating evidence indicates that certain output pathways of the circadian clock are not primarily controlled by target gene regulation, and several studies directly demonstrated posttranslational regulation of cellular processes by clock proteins. PER2, for example, has been shown to regulate DNA-damage signaling via association with the tumor suppressor tumor protein p53 (TP53) [8], while cryptochromes serve as cofactors for the E3-ligase complex SCF^FBXL3^, thereby linking the circadian clock to cell cycle control [9]. Such findings, together with a large-scale study of circadian protein-protein networks, lend support to the hypothesis that interactions of clock proteins with other cellular proteins could contribute significantly to the regulation of circadian output functions [10].

In this study, we address the role of the PER complex in the posttranslational regulation of GAPVD1, a regulator of small GTPases that has been identified as a substoichiometric component of cytoplasmic PER complexes in mice [3]. Knockdown of GAPVD1 lengthens the free running period of the molecular oscillator [3]**,** but nothing is known about the consequences of the association of GAPVD1 with the PER complex for the regulation of GAPVD1. Our results show that the control of CSNK1D-dependent GAPVD1 phosphorylation is a novel function of the PER complex that is linked to GAPVD1 degradation and that PER2 regulates GAPVD1 phosphorylation through a C-terminal autoinhibitory domain of CSNK1D.

## 2. Results

**Figure 1:**
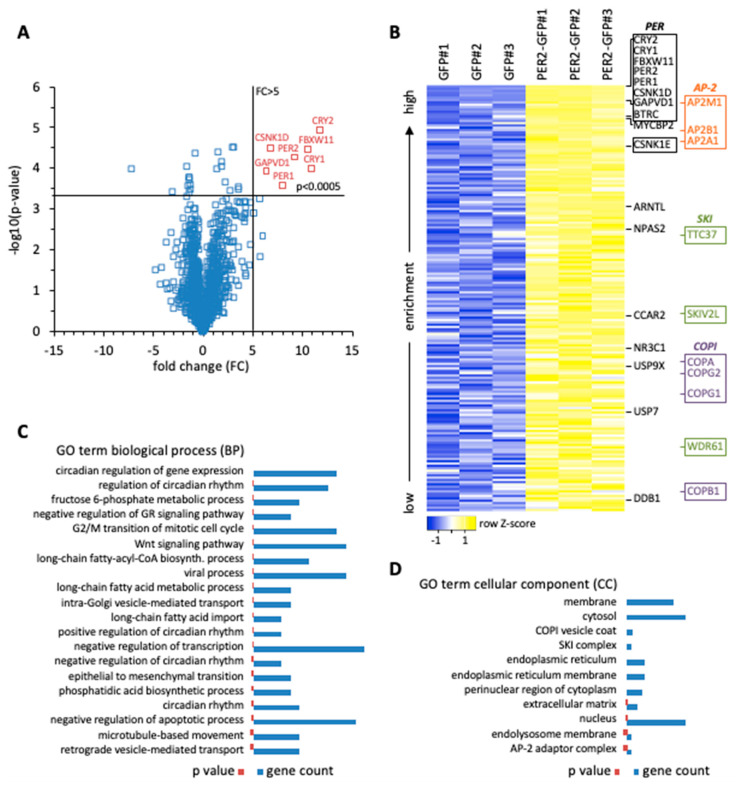
Affinity liquid chromatography–mass spectrometry (LC-MS) and gene ontology (GO) enrichment analyses of Repressor protein period 2 (PER2)-interacting proteins in human HT1080 cells. (**A**) Volcano plot of all proteins identified by affinity LC-MS. Enrichment is plotted as fold change (FC) against -log10(*p*-value) (*t*-test, n = 3). Crosslines delimit proteins with FC > 5 and *p* < 0.0005 highlighted in red; (**B**) Heat map of significantly enriched PER2-interacting proteins from control (GFP#1–3) and bait (PER2-GFP#1-3) purification replicates (FC > 0, *p* < 0.05). Colors indicate the distance of a signal from the sample mean in standard deviations (Z-score). Boxes highlight protein complexes. The heat map was created using heatmapper software [18]; (**C**,**D**) GO analysis of PER2-interacting proteins purged for tubulins, proteasomal, and ribosomal proteins, which are highly abundant in cells and often create an unspecific background in LC-MS experiments. The revised protein list was analyzed with DAVID [19] for the enrichment of GO terms in the categories biological process (BP) (**C**) and cellular component (CC) (**D**). The lists are sorted by increasing *p*-value shown as red bars (range: BP 1.2 × 10^−8^ − 0.004; CC 1.3 × 10^−10^ − 0.004). The number of genes in each group (gene count) is shown as blue bars (range: BP 3 − 12; CC 3 − 58).

Initially, we performed a biochemical screen for PER2-interacting proteins to advance our understanding of the transcription-independent roles of PER2. Whole cell extracts of human HT1080 fibrosarcoma cells stably expressing PER2-GFP were used for affinity enrichment with GFP-specific nanobodies. Proteins associated with PER2-GFP were analyzed by mass spectrometry, and enrichment was determined relative to cells expressing GFP alone (Supplementary Table S1). One group of proteins showed enrichment characteristics very similar to PER2 (fold change >5; *p* < 0.0005) (Figure 1A). This group contained, with the exception of PER3, all the proteins that were found to be part of cytoplasmic PER complexes in mice, i.e., PER1/2, CRY1/2, and CSNK1D, confirming that PER2-associated proteins were enriched with high specificity. Similar to the findings in mouse tissue [3], we identified GAPVD1 in human cells with the same enrichment characteristics as the other core clock proteins. Furthermore, the same group contained the E3 ligase F-box and WD repeat domain containing 11 (FBXW11/β-TrCP2), which marks clock proteins for degradation [11]. Less enriched, the core clock proteins beta-transducin repeat containing E3 ubiquitin protein ligase (BTRC/β-TrCP1), CSNK1E, ARNTL/BMAL1, and NPAS2, and the clock related proteins MYC binding protein 2 (MYCBP2) [12], nuclear receptor subfamily 3 group C member 1 (NR3C1) [13], ubiquitin specific peptidase 9 X-linked (USP9X) [14], ubiquitin specific peptidase 7 (USP7) [15], cell cycle and apoptosis regulator 2 (CCAR2) [16], and damage specific DNA binding protein 1 (DDB1) [17] were identified (Figure 1B).

Classification by gene ontology expectedly revealed that PER2-interacting proteins are mostly involved in the regulation of gene expression (Supplementary Table S2). Among gene regulatory processes, clock-related GO terms of biological processes were most numerous and ranked highest when sorted by increasing *p*-value (Figure 1C). They were followed by negative regulation of the glucocorticoid signaling pathway, which is a direct output pathway of the circadian clock [13], G2/M transition of the mitotic cell cycle, WNT signaling pathway, and negative regulation of transcription (Figure 1C). Additionally, the interaction of PER2 with all three components of the exosome-associated SKI complex was detected, in line with the finding that post-transcriptional mRNA regulation plays a significant role in determining the phase of rhythmic transcripts (Figure 1B,D), [20,21]. PER2 also interacted with key enzymes of metabolic pathways. The GO analysis pointed towards a regulatory node for channeling fructose 6-phosphate into either glycolysis or the hexosamine pathway (Figure 1C), and both pathways have been connected to the circadian clock. The hexosamine pathway provides the substrate UDP-GlcNAc for the posttranslational modification O-GlcNAcylation, which regulates clock protein function in the molecular oscillator [22]. The rate-limiting enzyme in the hexosamine pathway is Glutamine-fructose-6-phosphate transaminase (GFPT1/2), which was found to interact with PER2 (Supplementary Table S1). Alternatively, fructose 6-phosphate can enter the glycolytic pathway via Phosphofructokinase (PFKM/P/L), which is under circadian control and also interacted with PER2 (Supplementary Table S1), [23]. Furthermore, uptake and activation of long chain fatty acids might be regulated by direct interaction of transporter and ligase enzymes with PER2, as well as the biosynthesis of phosphatidic acid (Figure 1C).

It also became evident that a number of PER2-interacting proteins are involved in vesicular transport processes. The GO analysis identified intra-Golgi vesicle-mediated transport and retrograde vesicle-mediated transport involving the COPI coatomer, of which four out of seven core subunits were associated with PER2 (Figure 1B,D), [24]. In addition, three out of five components of the AP-2 adaptor complex were identified and are part of the GO terms microtubule-based movement and AP-2 adaptor complex (Figure 1B–D), [25]. Among them adaptor related protein complex 2 subunit mu 1 (AP2M1) ranked very high in the analysis, immediately following GAPVD1, and with a 6-fold enrichment at a *p*-value of 0.005, just missing the enrichment characteristics of the core PER complex components (Figure 1A and Supplementary Table S1). Importantly, GAPVD1 itself has a role in vesicular transport [26], which is mediated at least in part by binding to the AP-2 complex and regulating AP2M1 phosphorylation [27].

**Figure 2:**
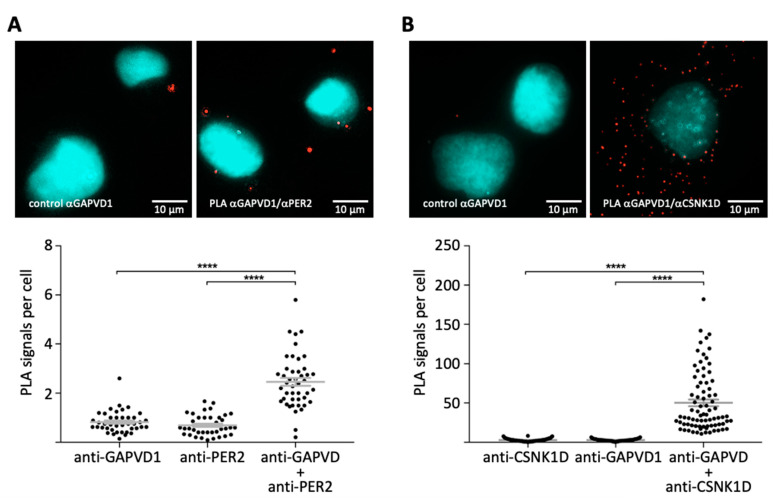
In situ detection of GTPase-activating protein and VPS9 domain-containing protein 1 (GAPVD1)-PER2 and GAPVD1-casein kinase 1 delta (CSNK1D) protein complexes by proximity ligation assay (PLA). (**A**) Immunofluorescence microscopy overlay images of PLA with primary antibodies against GAPVD1 and PER2 or (**B**) GAPVD1 and CSNK1D in wild type HT1080 cells. Red: PLA signals; cyan: DAPI. Lower panels: Quantification of PLA, including controls with only one primary antibody each. Data points represent single cells on at least five different microscopy slides in three independent experiments; **** *p* < 5 × 10^−5^ (*t*-test).

**Figure 3:**
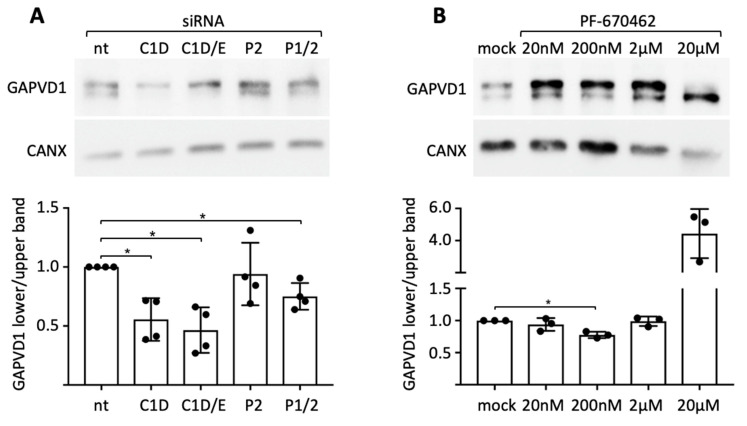
Downregulation of the PER complex components or inhibition of CSNK1D/E kinase activity modulates GAPVD1 phosphorylation levels in HeLa cells (**A**) Western blot analysis of GAPVD1 after treatment with siRNAs against CSNK1D (C1D), CSNK1D/E (C1D/E), PER2 (P2), PER1/2 (P1/2), or non-target siRNA (nt) in HeLa cells; (**B**) Western blot analysis of GAPVD1 after treatment of HeLa cells with increasing concentrations of PF670462 for 18 h. An antibody against Calnexin (CANX) was used as a control for equal loading. Lower panels: Quantification of GAPVD1 protein levels shown as the ratio between the faster (lower) and slower (higher) migrating GAPVD1 protein band normalized to cells treated with nt siRNA or mock-treated cells. Circles show single data points; error bars show standard deviations. * *p* < 0.05 (*t*-test).

So far, GAPVD1 has been connected to CSNK1D and PER complexes exclusively in vitro through affinity purification experiments (Figure 1A,B), [3,28]. To confirm that GAPVD1 also interacts with the PER complex in situ, we employed the proximity ligation assay in cultured human cells [29]. Fixed HT1080 cells were incubated with the antibody combinations GAPVD1/PER2, or GAPVD1/CSNK1D, or with only one of each antibody as a control. Subsequently, the samples were incubated with species-specific secondary antibodies coupled to DNA-oligonucleotides, which can only be ligated to a closed circle and used as a template in a rolling circle amplification reaction if the primary target proteins are located within a distance of less than ~30 nm from each other [29]. Amplified DNA was then hybridized to a sequence-specific fluorescent probe and visualized by fluorescence microscopy. Both antibody combinations GAPVD1/PER2 (Figure 2A) and GAPVD1/CSNK1D (Figure 2B) resulted in the formation of predominantly cytoplasmic fluorescent signals, whereas almost no signals were created by incubation with only one primary antibody. This shows that also in human cells, GAPVD1 forms protein complexes with PER2 and CSNK1D with a distance of less than 30 nm between GAPVD1 and its interaction partners, which is in good agreement with the reported size of ~25 nm for GAPVD1-containing cytoplasmic PER complexes in mouse liver [3].

Since the kinase activity of CSNK1D towards PER2 and other clock proteins is a cardinal function of the PER complex [3,30], and we have demonstrated a close association of CSNK1D and PER2 with GAPVD1 in situ (Figure 2), we asked if CSNK1D and PER2 are involved in the regulation of GAPVD1 phosphorylation. First, siRNA-mediated knockdown was employed in HeLa cells, in which two differently phosphorylated forms of GAPVD1 can be distinguished by their migration behavior on SDS gels (Supplementary Figure S1) [31]. Knockdown of CSNK1D strongly reduced the relative amount of the faster migrating form of GAPVD1, whereas additional knockdown of CSNK1E only slightly enhanced this effect (Figure 3A). Knockdown of both PER1 and PER2 but not of PER2 alone had the same effect as knockdown of CSNK1D, albeit to a lesser extent. Both CSNK1D and PER proteins seem, therefore, to be required for establishing the phosphorylation state of GAPVD1. Likewise, treatment of HeLa cells with the CSNK1D/E-specific kinase inhibitor PF670462 at a concentration of 200 nM decreased the relative amount of the faster migrating form of GAPVD1 (Figure 3B). Application of a hundredfold higher concentration of the inhibitor additionally reduced the amount of the higher phosphorylated form of GAPVD1, which might be explained by partial and full inhibition of CSNK1D at different concentrations. We conclude that the kinase activity of CSNK1D plays an essential role in setting the phosphorylation level of GAPVD1. The requirement for either PER1 or PER2 in this process demonstrates that CSNK1D-dependent phosphorylation of GAPVD1 is a function of the PER complex.

**Figure 4:**
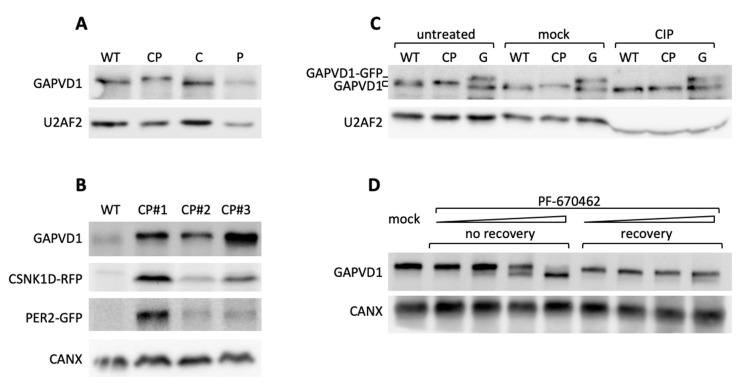
CSNK1D-dependent GAPVD1 phosphorylation is regulated by PER2 (**A**) Western blot analysis of GAPVD1 in HT1080 wild type cells, HT1080 cells overexpressing CSNK1D-GFP (C), PER2-GFP (P) or PER2-GFP and CSNK1D-RFP (CP) were analyzed with an antibody against human GAPVD1; (**B**) Clonal CP cells (CP#1–3) were analyzed with antibodies against GAPVD1, RFP, and GFP; (**C**) HT1080 wild type, and CP cells were analyzed untreated, mock-treated (phosphatase buffer, no calf intestinal phosphatase (CIP)) or CIP-treated (phosphatase buffer and CIP) and analyzed with an antibody against GAPVD1. HT1080 cells overexpressing GAPVD1-GFP were included as size marker; (**D**) CP cells were treated with increasing amounts (20 nM, 200 nM, 2 µM, 20 µM) of PF670462 for 18 h. Cells were harvested directly after treatment or allowed to recover for 24 h after withdrawal of the drug and analyzed with an antibody against GAPVD1. Antibodies against U2 small nuclear RNA auxiliary factor 2 (U2AF2) and Calnexin (CANX) were used as a control for equal loading.

Next, we addressed the requirements for GAPVD1 phosphorylation by protein overexpression in HT1080 cells, which in contrast to HeLa cells, have very low or undetectable levels of highly phosphorylated GAPVD1. GAPVD1 was analyzed by Western blot in cell lines stably expressing PER2-GFP (P), CSNK1D-GFP (C), or PER2-GFP and CSNK1D-RFP (red fluorescent protein) (CP). In CP cell lines that overexpress both proteins the GAPVD1 protein band migrated considerably slower than in wild type cells or cells that overexpress only PER2 or CSNK1D (Figure 4A). The effect was not specific for a single clonal cell line but occurred in several independently isolated CP cell lines with varying PER2-GFP and CSNK1D-RFP expression levels (Figure 4B). The altered migration behavior of GAPVD1 in CP cells was caused by protein phosphorylation since it was fully reverted by treatment of the cell extracts with calf intestinal phosphatase (CIP) before Western blot analysis (Figure 4C) and treatment of CP cells with PF670462 almost completely blocked GAPVD1 phosphorylation (Figure 4D). CSNK1D-dependent GAPVD1 phosphorylation and dephosphorylation are dynamic processes in the cell since high phosphorylation levels were fully restored after removing the inhibitor (Figure 4D).

**Figure 5:**
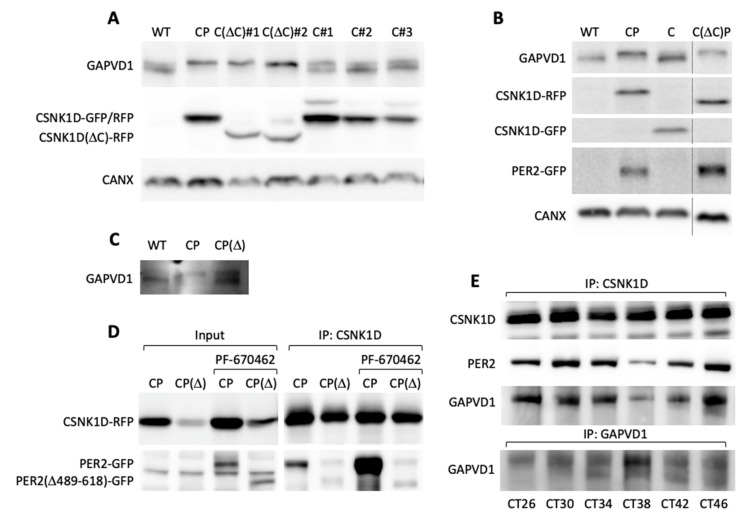
Phosphorylation of GAPVD1 is inhibited by the C-terminal tail of CSNK1D and requires direct interaction of CSNK1D and PER2. (**A**) Western blot analysis of GAPVD1 in HT1080 wild type cells, CP cells, and different clonal cell lines expressing CSNK1D(Δ317-342)-RFP (C(ΔC)#1-2) or CSNK1D-RFP (C#1–3); (**B**) HT1080 wild type cells and cells overexpressing CSNK1D-RFP or CSNK1D(Δ317–342)-RFP together with PER2-GFP (CP and C(ΔC)P), and cells overexpressing CSNK1D-GFP alone (**C**) were analyzed with antibodies against GAPVD1, CSNK1D, and PER2; an antibody against Calnexin (CANX) was used as a control for equal loading; dashed lines indicate lane splicing within one and the same Western blot membrane; (**C**) Western blot analysis of GAPVD1 in HT1080 wild type cells and cells expressing CSNK1D-RFP together with PER2-GFP (CP) or PER2(Δ489–618)-GFP (CP(Δ)); (**D**) CSNK1D was immunoprecipitated from CP or CP(Δ) cells with or without pre-treatment with PF-670462 for 18 h. Cell lysates (Input) and immunoprecipitated proteins were analyzed with antibodies against CSNK1D and PER2; (**E**) CSNK1D or GAPVD1 were immunoprecipitated from synchronized U2OS cells at the indicated circadian times (CT) after dexamethasone treatment, and immunoprecipitated proteins were analyzed with antibodies against CSNK1D, PER2, and GAPVD1. Similar levels of the non-rhythmic CSNK1D protein indicate equal IP efficiencies in all samples.

In combination, the knockdown and overexpression experiments strongly indicated a requirement for both CSNK1D and PER proteins for the establishment of GAPVD1 phosphorylation. At this point, two major questions seemed to arise. First, why is CSNK1D necessary but not sufficient for GAPVD1 phosphorylation? And second, which mechanistic role do PER proteins play in this process? It had been shown previously that the kinase activity of CSNK1D is inhibited by autophosphorylation of its C-terminal tail. More specifically, deletion of a region between Histidine 317 and Proline 342, which contains five serines and one threonine, creates a mutant enzyme with 10-fold increased activity [32]. Stable expression of this mutant C(ΔC) in HT1080 cells resulted in increased GAPVD1 phosphorylation levels in independent clonal cell lines, whereas expression of wild type CSNK1D alone did not lead to increased GAPVD1 phosphorylation relative to wild type cells (Figure 5A). Moreover, deletion of the autoinhibitory region fully relieved the PER2-dependence since co-expression of PER2 did not further enhance GAPVD1 phosphorylation levels in contrast to cells expressing wild type CSNK1D (Figure 5B).

These results suggest that PER2 stimulates the CSNK1D-dependent phosphorylation of GAPVD1 by counteracting the autoinhibitory activity of the C-terminal domain of CSNK1D, most likely via direct interaction with CSNK1D in the context of the PER complex. Indeed, deletion of an essential part of the casein kinase interaction domain in the PER2 mutant PER2(Δ489–618) [33] resulted in significantly lower levels of phosphorylated GAPVD1 when PER2(Δ489–618) was stably co-expressed with CSNK1D in HT1080 cells (Figure 5C). We verified that the binding of PER2(Δ489–618) to CSNK1D was strongly reduced compared to wild type PER2 (Figure 5D). Even treatment with PF670462, which stabilized both wild type and mutant PER2 [34], only enhanced the binding of PER2 but not of PER2(Δ489–618) to CSNK1D (Figure 5D). Moreover, in synchronized human U2OS osteosarcoma cells, which, in contrast to HeLa and HT1080 cells, possess a functional circadian oscillator, GAPVD1 was rhythmically associated with CSNK1D, and the strength of this interaction mirrored the binding of PER2 to CSNK1D (Figure 5E). Interestingly, association with the PER complex seemed to confer rhythmicity to GAPVD1 phosphorylation levels as judged by the changing intensity ratio of the upper and lower GAPVD1 bands during the 24 h time course (Figure 5E).

**Figure 6:**
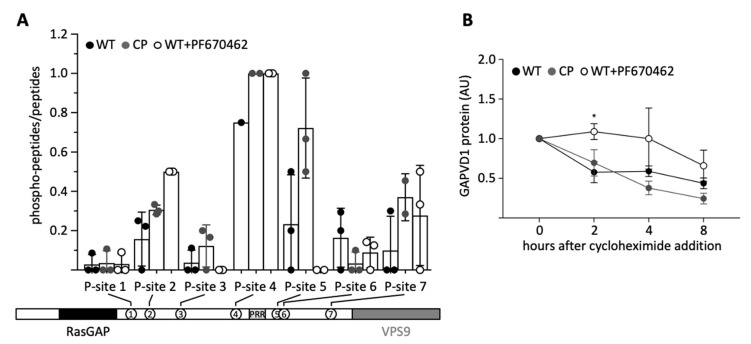
Phosphorylation regulates the speed of GAPVD1 degradation. (**A**) The ratio of phosphorylated to unphosphorylated peptides in each sample is shown for all peptides that were identified in HT1080 wild type cells (black), CP cells (grey), or wild type cells pre-treated with 20 µM PF670462 for 18 h (white). Circles show single data points; error bars show standard deviations. Samples in which neither a phosphorylated nor an unphosphorylated peptide was detected for a specific phosphorylation site (P-site) were omitted from the dataset. The locations of P-sites relative to the RasGAP and VPS9 domains and a proline rich region (PRR) in GAPVD1 are shown underneath [26]; (**B**) Degradation kinetics of GAPVD1 after treatment with 50 µM cycloheximide in HT1080 wild type cells (black), CP cells (grey) or wild type cells pre-treated with 20 µM PF670462 for 18h (white). GAPVD1 protein levels were normalized to Calnexin, and t = 0 values were set to 1. Lines connect replicate means (n = 4); error bars show standard errors of mean. * *p* < 0.05 (*t*-test).

Since GAPVD1 is phosphorylated by CSNK1D as part of the PER complex, and CSNK1D-dependent phosphorylation at two antagonistic sites regulates the degradation of PER2 in a mechanism called the phosphoswitch [35], we asked if the phosphorylation state of GAPVD1 also regulates its stability. First, we characterized several differentially phosphorylated forms of GAPVD1 by affinity-purification of endogenous GAPVD1 from HT1080 wild type cells, CP cells, and wild type cells treated with 20 µM PF670462. GAPVD1 phosphopeptides were quantified by mass spectrometry. The sequence coverage was similar under all conditions (WT 42.7 ± 7.6%; CP 41.3 ± 10.3%; WT + PF670462 38.7 ± 7.0%; mean ± standard deviation; n = 3), and in total seven different phosphopeptides were identified (Figure 6A). All phosphorylation sites mapped to the central part of GAPVD1 outside the RasGAP and VPS9 domains. Compared to untreated wild type cells, phosphorylation levels at site 5, and less pronounced at site 3, were higher in CP cells and lower after inhibitor treatment. In contrast, phosphorylation levels at all other sites did not show a consistent response to increased or decreased CSNK1D activity. For example, site 4 was highly phosphorylated under all conditions, and phosphorylation at site 2 was highest in PF670462-treated cells. Phosphorylated amino acids within the peptides could be predicted with a certain confidence (Supplementary Table S3). Site 3 contains the casein kinase consensus site pS-X-X-S (underlined is the target site for phosphorylation) [36], which might explain its dependence on CSNK1D activity. However, this motif is also present in the CSNK1D-independent site 4 (Supplementary Table S3). Moreover, casein kinase 1 family members, including CSNK1D, have been shown to efficiently phosphorylate a large number of non-consensus sites [30]. Primary sequence determinants alone are, therefore, unlikely to explain the variable dependence on CSNK1D activity at the different sites, and our results do not rule out that sites 3 and 5 are additionally phosphorylated by other kinases.

Finally, we examined the degradation kinetics of GAPVD1 after blocking translation with cycloheximide under the same conditions that were used for the phosphopeptide analysis, i.e., in wild type, CP, and PF670462-treated cells, all of which have distinguishable GAPVD1 phosphorylation patterns (Figure 6A). Treatment of wild type cells with PF670462 significantly delayed the degradation of GAPVD1 (Figure 6B). Two hours after cycloheximide treatment, no decrease in GAPVD1 levels was observed in PF670462 treated cells, whereas in untreated cells, GAPVD1 levels had fallen to ~50%. Hence, the speed of GAPVD1 degradation is affected by blocking CSNK1D/E-dependent phosphorylation. In contrast, the speed of GAPVD1 degradation in CP cells was similar to that in untreated wild type cells (Figure 6B), indicating that phosphorylation destabilizes GAPVD1 in wild type cells and is not further enhanced by increasing CSNK1D-dependent phosphorylation levels.

## 3. Discussion

GAPVD1 is linked to the molecular circadian oscillator as a component of cytoplasmic PER complexes, but little is known about the functional consequences of this interaction. It has been suggested that GAPVD1 might play a role in the assembly, accumulation, trafficking, or nuclear entry of PER complexes, since knockdown of GAPVD1 lengthens the circadian period of cultured cells and GAPVD1 must, therefore, interfere with the molecular clockwork [3]. Our study extends this hypothesis by providing evidence that the PER complex can impact the function of GAPVD1 by regulating GAPVD1 phosphorylation. We found in the loss of function experiments that both CSNK1D and PER proteins are required to establish the phosphorylation state of GAPVD1, and since all PER1/2 molecules in the cell are incorporated into PER complexes [3], CSNK1D-dependent phosphorylation of GAPVD1 must be to a significant part a function of PER complexes. However, our results do not rule out that phosphorylation of GAPVD1 by CSNK1D might also take place outside of PER complexes. Indeed, CSNK1D is known to phosphorylate GAPVD1 in other cellular contexts, although in these cases, participation of PER complexes cannot be excluded [31,37]. GAPVD1 is also likely to be phosphorylated by additional kinases [38] since phosphorylation is not fully blocked by inhibition of CSNK1D/E. Both mechanisms could still be connected if CSNK1D activated other kinases through phosphorylation or modified GAPVD1 with priming or inhibitory phosphorylation marks for other kinases.

How do PER proteins regulate the phosphorylation of GAPVD1? Our data indicates that PER2 can relieve CSNK1D from the effect of an autoinhibitory domain in its C-terminus. Unlike many other kinases, CSNK1D is not regulated by phosphorylation of its activation loop [39]. Its activity is controlled rather by anionic binding molecules, such as heparin or stretches of phosphorylated amino acids in proteins, which counteract the activity of the autoinhibitory domain [32] and facilitate a conformational change in CSNK1D that alters its target specificity [30]. In the well understood phosphoswitch mechanism, this results in CSNK1D favoring phosphorylation of the degron site over the FASP (familial advanced sleep phase) site in PER2. For CSNK1D-dependent GAPVD1 phosphorylation, a similar mechanism might exist, in which phosphorylated PER2 works as the anionic binding molecule that directs the enzymatic activity of CSNK1D towards specific phosphorylation sites in GAPVD1. It is also conceivable that the tight association of PER2 with CSNK1D through the casein kinase binding domain in PER2 [40] facilitates capturing of the autoinhibitory domain of CSNK1D by PER2. In line with our observations, deletion of PER proteins affects substrate selectivity in the nuclear PER complex [3], and PER2 shifts the activity of CSNK1D away from output proteins towards core clock proteins [41], which should include GAPVD1 as a PER complex component. Activation of casein kinases by interacting proteins has also been found in the WNT signaling pathway, where phosphorylation of Frizzled 6 (FZD6) by CSNK1E requires the protein dishevelled homolog (DVL) [42].

Another question concerns the biological role of GAPVD1 phosphorylation. We show here that the phosphorylation state of GAPVD1 affects the speed of its degradation, which might affect the function of GAPVD1 as a regulator of the molecular oscillator, for example, by delaying or promoting the nuclear entry of the PER complex. However, our experiments do not allow any conclusions about the direct effects of phosphorylation by the PER complex on GAPVD1 degradation. Nevertheless, in analogy to the phosphoswitch model, we found that total phosphorylation levels seem to be less important for the degradation kinetics than phosphorylation of individual sites in GAVPD1. In particular, phosphorylation of site 5 markedly responded to the level of CSNK1D kinase activity, and degradation was strongly delayed when site 5 was fully dephosphorylated. Interestingly, a comprehensive study of the circadian phosphoproteome found site 5 to be rhythmically phosphorylated [43] and to be a target for AMP-activated protein kinase (AMPK) activity [38], which itself has a diurnal rhythm [44].

There is also evidence that phosphorylation of GAPVD1 has a role in vesicle mediated transport processes [31]. Initially, GAPVD1 was found in a screen for regulators of endocytosis in *Caenorhabditis elegans*, a function attributed to its C-terminal VPS9-domain, which acts as a GEF (Guanine nucleotide exchange factor) for small GTPases of the Ras-related in brain 5 (RAB5) family [45]. The function of its N-terminal RasGAP domain is largely unknown but might involve a role in post-endocytic degradation mechanisms through the recruitment of ubiquitin ligases [46]. It is unclear, however, if these domains are directly regulated by CSNK1D-dependent phosphorylation since we and others have found GAPVD1 to be phosphorylated mostly in the central part of the protein [31,37]. Nevertheless, our affinity enrichment analysis shows that GAPVD1 is only one of a number of proteins involved in vesicular transport that is associated with PER2, which creates a speculative link between PER complexes and vesicular transport processes. Ample evidence exists already that the circadian clock controls vesicular transport. We have previously shown that autophagy is regulated by the circadian clock [47]. Control of the secretory pathway by the circadian clock has been demonstrated in the liver [48], shown to be linked to rhythmic collagen synthesis and degradation [49] and to regulate vesicle cycling in the suprachiasmatic nucleus [50] as well as exocytosis in pancreatic cells [51]. Future studies might address the role of GAPVD1 phosphorylation in vesicular transport linked to control of the molecular oscillator and output functions of the clock.

## 4. Materials and Methods

### 4.1. Cell Lines

HT1080 and HeLa cells were purchased from the German Collection of Microorganisms and Cell Cultures (DSMZ). Stable cell lines were generated by Effectene (301427, Qiagen, Hilden, Germany) transfection of HT1080 cells. One microgram plasmid-DNA was used in a T25 cell culture flask; antibiotic containing selection medium was added after 48 h, and single clones were isolated after 10–30 days. The resulting cell lines were HT1080-PER2-GFP (P); HT1080-GAPVD1-GFP (G); HT1080-CSNK1D-GFP (C); HT1080-GFP; HT1080-CSNK1D-RFP-PER2-GFP (CP); HT1080-CSNK1D(D317-342)-RFP (C(DC)). All cell lines were cultured in Dulbecco’s modified Eagle’s medium (DMEM, Gibco, Thermo Fisher Scientific, USA) supplemented with 10% FCS, 100 U/mL penicillin, 100 µg/mL streptomycin and, depending on the cell line, 0.4 µg/mL puromycin or 300 µg/mL hygromycin, and maintained at 37 °C in a 5% CO_2_ environment.

### 4.2. Antibodies

GAPVD1/RAP6 (NBP1-19156, Novus Biologicals, Centennial, CO, USA); GAPVD1/RAP6 in Duolink (SAB1401626, Sigma, St. Louis, MO, USA); PER2 (20359-1-AP, Proteintech, Rosemont, IL, USA); CSNK1D (ab85320, Abcam, Cambridge, UK); CyTM3 Anti-Mouse IgG Antibody (115-165-205, Jackson ImmunoResearch, West Grove, PA, USA); CyTM3 Anti-Rabbit IgG Antibody (111-166-003, Jackson ImmunoResearch, West Grove, PA, USA); ECLTM Anti-Mouse IgG Antibody (NA931V, GE Healthcare, Chicago, IL, USA); ECLTM Anti-Rabbit IgG Antibody (NA934V, GE Healthcare).

### 4.3. Affinity Enrichment

For affinity enrichment of GFP from HT1080-PER2-GFP and HT1080-GFP cell lines, cells were lysed in CHAPS lysis buffer (20 mM Tris HCl pH 7.5, 150 mM NaCl, 0.03% CHAPS, 0.5 mM EDTA, 10 µM Na_2_MoO_4_, 1mM Na_3_VO_4_, 10 mM NaF, 2.5 mM Na_4_P_2_O_7_, 1x protease inhibitor cocktail (PI) (Roche, Basel, Switzerland)). Twenty-five milligrams of cleared whole cell protein extract was incubated with 40 µL equilibrated GFP-Trap^®^ beads (ChromoTek, Martinsried, Germany) with rotation at 4 °C overnight. Thereafter, the beads were washed three times with 1 mL CHAPS lysis buffer and then three times with high salt CHAPS lysis buffer (20 mM Tris HCl pH 7.5, 500 mM NaCl, 0.03% CHAPS, 0.5 mM EDTA, 10 µM Na_2_MoO_4_, 1 mM Na_3_VO_4_, 10 mM NaF, 2.5 mM Na_4_P_2_O_7_, 1X PI (Roche)). The beads were suspended in 60 µL Buffer A (50 mM Tris HCl pH 7.5, 0.1 mM EGTA) and then subjected to LC-MS or Western blot analysis. For affinity enrichment of GAPVD1 from HT1080 and HT1080-CSNK1D-RFP-PER2-GFP cell lines, 10^7^ cells were lysed in Triton X-100 lysis buffer (150 mM NaCl, 1% Triton X-100, 50 mM Tris HCl pH 8.0, 1X PI (Roche), 1X phosphatase inhibitor cocktail (Roche). Thereafter, the cleared cellular lysates were incubated with 120 µL Protein A microbeads (Miltenyi Biotec, Bergisch-Gladbach, Germany) and 30 µL GAPVD1 antibody (Novus Biologicals) with rotation at 4 °C overnight. Subsequently, the cell lysates were applied on equilibrated m Column (Miltenyi Biotec) that were placed in the magnetic field of the m MACS™ Separator (Miltenyi Biotec). The columns were washed 4 times with high salt lysis buffer (500 mM NaCl, 1% NP-40, 50 mM Tris HCl pH 8.0, 1X PI (Roche), 1X phosphatase inhibitor cocktail (Roche)) and then one time with low salt lysis buffer (1% NP-40, 50 mM Tris HCl pH 8.0, 1X IP (Roche), 1X phosphatase inhibitor cocktail (Roche)). Proteins were eluted with 40 µL preheated (95 °C) SDS gel loading buffer (50 mM Tris HCl pH 6.8, 50 mM DTT, 1% SDS, 0.005% bromophenol blue, 10% glycerol, 1X PI (Roche), 1X phosphatase inhibitors cocktail (Roche)) and then subjected to LC-MS. For CSNK1D inhibitor treatment, cells were incubated for 18h with 20 µM PF-670462 before lysis.

### 4.4. Sample Preparation for Mass Spectrometry

Proteins were separated by SDS PAGE, and isolated gel pieces were reduced with 50 µL 10 mM DTT, alkylated with 50 µL 50 mM iodoacetamide, and subjected to tryptic digestion with 6 µL 200 ng trypsin, 100 mM ammonium bicarbonate. The peptides were resolved in 15 µL 0.1% trifluoracetic acid and subjected to liquid chromatography.

### 4.5. LC-MS Analysis

For the LC-MS analysis, a QExactive plus (Thermo Scientific, Waltham, Massachusetts, MA, USA) connected to an Ultimate 3000 Rapid Separation liquid chromatography system (Dionex/Thermo Scientific) equipped with an Acclaim PepMap 100 C18 column (75 µm inner diameter, 25 cm length, 2 mm particle size from Thermo Scientific) was used. The length of the LC gradient was 60 min. The mass spectrometer was operating in positive mode and coupled to a nanoelectrospray ionization source. The capillary temperature was set to 250 °C and source voltage to 1.4 kV. In the QExactive plus mass spectrometer for the survey scans, a mass range from 200 to 2000 m/z at a resolution of 140,000 was used. The automatic gain control was set to 3,000,000, and the maximum fill time was 50 ms. The 10 most intensive peptide ions were isolated and fragmented by high-energy collision dissociation (HCD).

### 4.6. Computational Mass Spectrometric Data Analysis

Proteome Discoverer (version 2.3.0.523, Thermo Scientific) was used for peptide/protein identification with Mascot (version 2.4, Matrix Science, London, UK) as a search engine employing the human database (Uniprot, Swissprot). A false discovery rate of 1% (*p* ≤ 0.01) on the peptide level was set as the identification threshold.

### 4.7. Proximity Ligation Assay (Duolink)

Three to four times ten to the four cells were harvested and cultivated in removable 4-well µ-Slides (Ibidi, Martinsried, Germany) for 24 h at 37 °C. Cells were fixed and permeabilized with 4% formaldehyde for 10 min, followed by 0.1% Triton X-100 for 5 min at room temperature. Blocking was performed with 2 drops of Duolink blocking solution (Duolink In Situ Detection Reagents Red, Sigma) per well for 60 min at 37 °C. Thereafter, slides were incubated with primary antibodies diluted in Duolink antibody diluent at 4 °C overnight on an orbital shaker. After washing four times with wash buffer A, PLA probes were added (70 µL/well) for 1 h at 37 °C in a humid chamber. Ligation and amplification were performed according to the manufacturer’s instructions (Duolink In Situ Detection Reagents Red, Sigma). Slides were washed two times with wash Buffer B at room temperature for 10 min, then overnight at 4 °C. After washing, cells were stained with DAPI 1:1000 in PBS for 4 min, and slides were covered with slide slips. Pictures were taken with an Axiovert 100 microscope and analyzed with Image J.

### 4.8. In Vitro Dephosphorylation of Cell Extracts

Whole cell extracts of HT1080 cells lysed in 1× Laemmli buffer without SDS were sonicated and subsequently incubated at 65 °C for five minutes. The samples were cooled on ice and then treated with 1U Calf Intestinal Phosphatase (NEB, Ipswich, Massachusetts, USA) per microgram of protein in CutSmart buffer (NEB) for 30 min at 37 °C. After the dephosphorylation reaction, SDS was added to a final concentration of 1%, and proteins were separated by SDS-PAGE.

### 4.9. siRNA-Mediated Knockdown

Ten to the five cells were seeded on 6-well plates one day before siRNA transfection. siRNAs targeting CSNK1D (L-003478-01-0005, Dharmacon, Lafayette, CO, USA), CSNK1E (L-003479-00-0005, Dharmacon), or non-target (nt) control siRNA (D-001810-10-05, Dharmacon) were incubated with DharmaFECT 1 Transfection Reagent (T-2001-03, Dharmacon) according to the manufacturer’s instructions. Transfection was carried out at a concentration of 100 nM for each siRNA. Cells were collected after 48 h for mRNA analysis by qPCR or after 72 h for protein analysis by Western blotting.

### 4.10. Immunoprecipitation with Synchronized U2OS Cells

Two point five times ten to the six U2OS cells were seeded on T175 flasks two days before dexamethasone shock. Cell synchronization was performed with 100 nM dexamethasone (D4902, Sigma) for 30 min at 37 °C. Cells were collected after 26 h, 30 h, 34 h, 38 h, 42 h, and 46 h after dexamethasone treatment. Immunoprecipitations were performed from 4 mg whole cell protein extract with 4 µg CSNK1D (ab103955, Abcam) or 4 µg GAPVD1 antibody (NBP1-19156, Novus Biologicals).

## Data Availability

Data is contained within the article or supplementary material.

## Supplementary Materials

The following are available online at https://www.mdpi.com/article/10.3390/ijms22073787/s1, Figure S1: Knockdown efficiencies in Figure 3A, Table S1: LC-MS PER2-GFP, Table S2: LC-MS PER2-GFP GO analysis, Table S3: GAPVD1 P-sites.
