# Supplementary figures and images for: Phosphorylation of GAPVD1 Is Regulated by the PER Complex and Linked to GAPVD1 Degradation
Source: Int J Mol Sci. 2021 Apr 6;22(7):3787. doi: 10.3390/ijms22073787 (PMC8038846; doi:10.3390/ijms22073787)

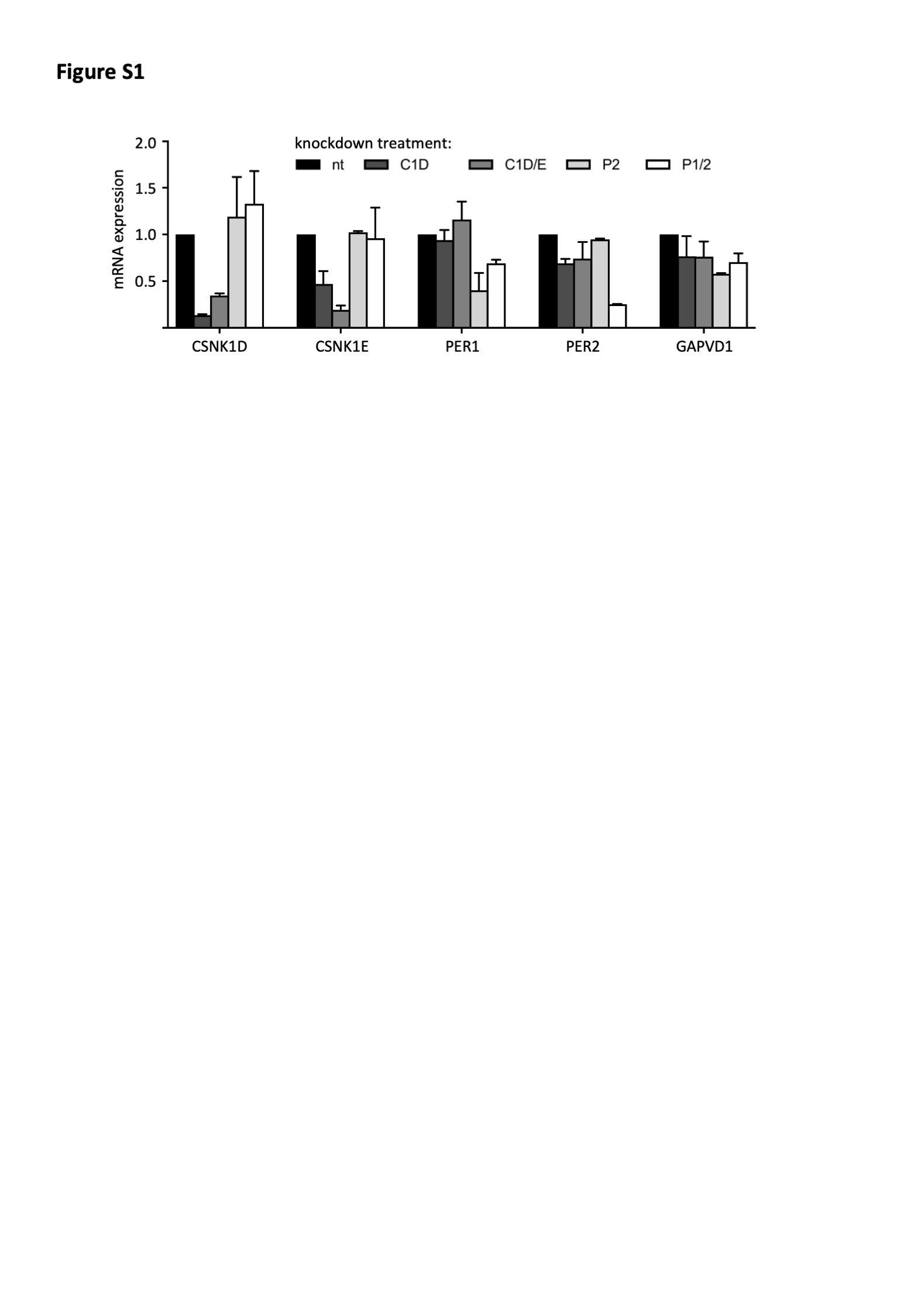

Supplement: Supplementary file 1 [file ijms-22-03787-s001.zip › Figure S1.tiff]
